# Osteopontin counters human immunodeficiency virus type 1–induced impairment of neurite growth through mammalian target of rapamycin and beta-integrin signaling pathways

**DOI:** 10.1007/s13365-019-00729-y

**Published:** 2019-02-13

**Authors:** Mathilde Calvez, George Hseeh, Simon Benzer, Amanda M. Brown

**Affiliations:** 1 Department of Biology, Ecole Normale Superieure de Lyon, Lyon, France; 2 Department of Neurology, Johns Hopkins University School of Medicine, 600 North Wolfe Street, Meyer 6-119, Baltimore, MD 21287 USA

**Keywords:** Cytoskeleton, HIV-associated neurocognitive disorder, Integrins, Dendrites, Neurons

## Abstract

Despite the fact that human immunodeficiency virus type 1 (HIV-1) does not enter or replicate in neurons, its infection of a subset of resident brain glia cells (microglia and astrocytes) induces via disparate mechanisms, dysregulation of glutamate metabolism, neurotoxicity, and inflammation. Antiretroviral therapies suppress viral load, but cellular activation and release of proinflammatory factors, some of which is likely related to viral reservoirs, continue to promote a microenvironment that is injurious to neurons. However, the molecular mechanisms remain to be identified. Osteopontin (OPN) is a proinflammatory cytokine-like, extracellular matrix protein that is elevated within the brain and CSF in several neurodegenerative disorders, including HIV-associated cognitive disorder. However, the impact of elevated OPN on neuronal integrity and function in HIV-infected individuals who exhibit cognitive dysfunction remains unknown. In this study, using a neuronal cell line and primary cultures of cortical rat neurons, we identify the mammalian target of rapamycin pathway involvement in a signaling interaction between OPN-β1-integrins and the HIV-1 envelope glycoprotein, which stimulates neurite growth. These findings link for the first time HIV X4-envelope receptor engagement and osteopontin-mediated signaling through β1-integrin receptors to the mTOR pathway and alterations in the cytoskeleton of cortical neurons.

## Introduction

There are approximately 36 million people worldwide living with human immunodeficiency virus type I (HIV-1) infection, a third of whom are on suppressive antiretroviral therapy (ART). At the early stage of infection, the virus invades the central nervous system (CNS) through infiltrating infected monocytes and T lymphocytes and virus particles, thereby infecting resident myeloid cells and astrocytes in the brain (Kraft-Terry et al. 2010). HIV propagation in the brain impairs neuronal function and is at the origin of a spectrum of executive function defects including motor, behavioral, and cognitive deficits, known collectively as HIV-associated neurocognitive disorders (HAND) (McArthur et al. 2010). HAND affects between 33 and 60% of infected individuals, and despite the use of ART, the prevalence of this comorbidity has increased. This increase is partly linked to the advancing age of those infected, and the incomplete suppression of immune activation and viral replication in the periphery as well as in the brain (Heaton et al. 2010; Heaton et al. 2011). The three categories under the HAND umbrella, which are defined through an extensive and time-intensive battery of neuropsychological tests, include the most severe form HIV-associated dementia (HAD), minor cognitive motor disorder (MCMD), and asymptomatic cognitive impairment (ANI) (Antinori et al. 2007). Although HAD is characterized in part by reactive astrocytosis and an increase of activated macrophages and resident microglia (Kaul et al. 2001), this disorder results mostly from intensive neuronal injury and apoptosis in several brain regions, including the frontal cortex, hippocampus, cerebellum, and striatum (Kaul 2008; Holt et al. 2012)*.* Because HIV does not infect neurons, which lack the CD4 receptor, indirect factors have been implicated in the development of HIV-induced neural dysfunction in the CNS. Some of these mechanisms involve the HIV proteins Env, Tat, and Vpr, while others include indirect mechanisms linked to the release of proinflammatory and neurotoxic molecules by infected or activated macrophages and microglia, which induce signaling cascades leading to axonal injury and defective synaptodendritic connections and culminating in the establishment of neuropsychiatric and cognitive impairment (Kaul et al. 2001; Ellis et al. 2007; Kraft-Terry et al., 2010)*.*

The HIV-1 envelope (Env) glycoprotein gp120 has been shown to cause neuronal damage and apoptosis both in vitro and in vivo through mechanisms that potentiate the activity of glutamate receptors, which can lead to neuronal excitotoxicity and the activation of caspases (Toggas et al., 1994, Hesselgesser et al., 1998, Meucci et al., 1998, Kaul et al., 2007). HIV Env is a major factor mediating neurotoxicity, which can be released by infected glia and macrophages and subsequently bind to chemokine receptors such as CXCR4 (X4) and CCR5 (R5). These receptors are also expressed by astrocytes and neurons. R5 strains of HIV-1 are the predominant species during the early stages of infection, whereas X4 strains appear later, after a switch in co-receptor usage, which occurs in about 50% of patients (Asjo et al., 1986, Conner et al., 1997). Despite the fact that HIV-1 does not enter or replicate in neurons, its infection of a subset of resident brain glia cells (microglia and astrocytes) induces via disparate mechanisms, dysregulation of glutamate metabolism, neurotoxicity, and inflammation. Although antiretroviral therapies suppress viral load, cellular activation and release of proinflammatory factors continue to promote a microenvironment that is injurious to neurons. These factors are likely related to viral reservoirs. Further, the molecular mechanisms remain to be identified. Collectively, we and others have shown that the multifunctional proinflammatory cytokine-like protein osteopontin (OPN) is elevated in the plasma, cerebrospinal fluid, and brain tissue of HIV-infected individuals with moderate to severe cognitive impairment (Burdo et al., 2007, Burdo et al., 2008, Brown et al., 2011, Marcondes et al., 2014). The source of OPN in the brain likely derives in part from its expression and release from cells of the myeloid, astrocytic, and neuronal compartments (Silva et al., 2015). However, the impact of elevated OPN on neuronal integrity and function in HIV-infected individuals who exhibit cognitive dysfunction remains unknown. While it has been shown that OPN can signal through pro-survival pathways, as well as exacerbate proinflammatory ones, its impact on the integrity and function of neurons in the context of health and disease is underexplored.

In this study, differentiated human neuroblastoma cells and rat primary cultured cortical neurons were used to ask whether the outcome of OPN signaling in these cells is protective or synergizes with X4-tropic HIV IIIB envelope–induced injury to these cells. Given that infection by R5-utilizing HIV predominates in the brain, Env from the HIV SF162 strain was included in the study. An unexpected protective impact on neurite growth was observed and a mechanistic link between β-integrin and mTORC1 and mTORC2 signaling pathways was identified.

## Materials and methods

### Methods

#### Cell culture

SHSY5Y cells (ATCC) were grown in Dulbecco’s Modified Eagle Medium (DMEM)/F12 50%, containing 10% fetal bovine serum, 1% penicillin/streptomycin (Pen/Strep), and 1% glutamine (ThermoFisher). Cells were split, and the media was changed every 3–4 days. Cells were then plated either on 24- or 12-well plates (Costar) in neurobasal media (NB) (supplemented with B-27 and 1% glutamine), at a density of 10^5^ cells/well and differentiated for 7 days with 10 μM retinoic acid (Kovalevich and Langford 2013). Primary rat cortical neurons from the pre-frontal cortex of E18 Sprague/Dawley or Fischer 344 (Neuromics) were prepared as suggested by the manufacturer and grown for 4 days in B27/NB media (ThermoFisher) in 24-well plates supplemented with 1% Pen/Strep and 0.5 mM glutamine.

#### Experimental treatments

Envs from HIV-1 BaL gp120, clade B; SF162 gp140 trimer, clade B; and IIIB rgp120 (CHO), clade B representing either R5 or X4 strains were obtained from the NIH AIDS Research and Reference Reagent Program, Division of AIDS, NIAID, NIH (catalog #4961, #11784) (Cheng-Mayer et al., 1989, Lopez de Castro 1989). Differentiated SHSY5Y cells were incubated for 48–72 h with OPN (50 to 200 ng/mL, catalog #1433-OP/CF, #6359-OP-050 R&D systems), either alone or with BaL or IIIB gp120 Env proteins, at different concentrations, from 50 to 400 pM, as indicated in the text. Reconstituted Envs were stored in aliquots to avoid repeated freeze-thaw and stored at − 80 °C. One set of differentiated cells was incubated in triplicate wells, with or without 400 pM HIV IIIB envelope for 48–72 h and increasing concentrations of OPN from 6.25 pM, 12.5 pM, 25 pM, 50 pM, 100 pM, or 200 pM and 20 nM rapamycin or vehicle control. The anti-β1-integrin antibodies (purified hamster anti-rat CD29 clone Ha2/5, #555003 BD Pharmingen) and anti-β3-integrin antibodies (hamster anti-integrin beta 3 clone 2C9.G3 #ab171216 Abcam) were used at 1–2 μg/ml.

#### Immunofluorescent staining

SHSY5Y cells were fixed for 15 min with 4% paraformaldehyde and permeabilized with 0.2% Triton X-100 for 5 min. Non-specific binding sites were blocked by incubation for 30 min with 10% goat serum in PBS, pH 7.4. To visualize axons, cells were incubated overnight at 4 °C with β-III-tubulin (#MAB1637, Millipore), at 1:100 dilution in PBS. After washing with PBS, cells were incubated for 1 h with secondary polyclonal antibodies, Alexa Fluor 568-conjugated goat anti-mouse (ThermoFisher), at 1:100, at room temperature. Cortical cultures were prepared for immunofluorescence in the same manner as above and in addition to β-III-tubulin stained for dendrites using an antibody against MAP2 (1:500, MO22116, Neuromics) and NF (axons) (1:100, #2837, Cell Signaling). Cells were washed then incubated for 1 h at room temperature with secondary polyclonal antibodies, Alexa Fluor 488 (#A11017), 568 (#A11077) (ThermoFisher), and with DAPI or Hoechst stain to visualize nuclei. Images from cells were acquired using a Zeiss Axio Observer.Z1 (Oberkochen, Germany) microscope using a 20×/0.35 or 40×/0.5 objective.

#### Western blot

Medium was removed, and cells were carefully washed with PBS before the addition of cold N-PER buffer (ThermoFisher) containing a cocktail of protease and phosphatase inhibitors (Cell Signaling #5872). Cell lysates were clarified by centrifugation at 4 °C and stored in aliquots at − 80 °C. NuPAGE 4–12% Bis-Tris gels (ThermoFisher) were loaded with 2 μg of sample protein per lane and ran at constant voltage for 1.5–2 h before blotting to either nitrocellulose or PVDF membrane using the iBLOT2 system (ThermoFisher). The antibodies used were anti-phospho-PDK1 (pSer241); anti-phospho-SGK (pSer422); anti-mTOR; anti-mTOR pSer2481 (SAB4504514, SAB4503834, SAB4501038, SAB4301526 Sigma-Aldrich); PDK1 (MA5–15797, ThermoFisher); SGK1; b-actin; anti-phospho-S6 ribosomal protein (Ser235/236) (#12103, #8457, #2211 cell signaling); and anti-p70 S6K (#05-781R, Millipore). Band intensities were quantified using ImageJ version 1.51 software (Schneider et al., 2012).

## Statistical analyses

### Statistical analyses

To evaluate the mean axonal length of axons in differentiated SHSY5Y cells after each treatment, five pictures were taken for each condition and 20 axons were measured on each image, using the measure tool of the AxioVision Rel. 4.8 software (Zeiss). For analyses of immunofluorescent staining, 6 to 16 pictures were taken for each condition using a Zeiss Axio Observer.Z1 microscope using a 20×/0.35 or 40×/0.5 objective. Quantification of fluorescent labeling and western band intensities was performed using ImageJ 1.51. Statistical analyses were performed either by one-way ANOVA and subsequent Tukey’s test for multiple comparisons or by Kruskal-Wallis test then Dunn’s test, using GraphPad Prism v 6–7. Data were analyzed as ± standard deviation (SD), and a minimum *p* value of 0.05 was estimated as the significance level for all tests. A digital copy of the raw image was adjusted in the same manner for each, for optimal brightness and contrast using Adobe Photoshop CS5.1.

## Results

### In contrast to HIV envelope from the CXCR4-utilizing strain HIV_IIIB_, gp120 from CCR5-using HIV_BaL_ or HIV_SF162_ promotes neurite growth in retinoic acid–differentiated SHSY5Y neuronal-like cells

**Fig. 1:**
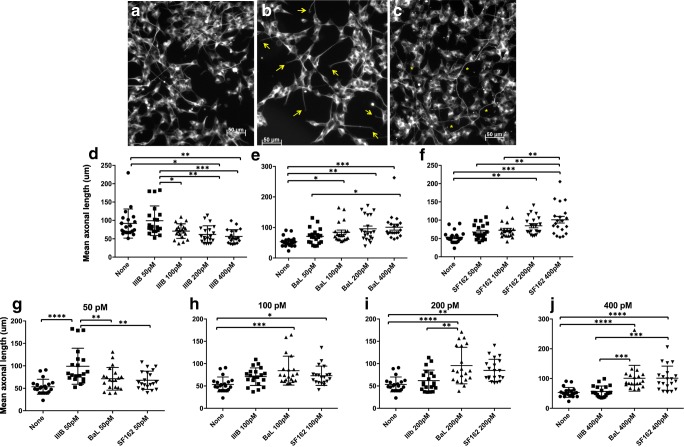
gp120 from CCR5-using HIV_BaL_ or HIV_SF162_, but not CXCR4-utilizing strain HIV_IIIB_, promotes neurite growth in retinoic acid–differentiated SHSY5Y neuronal-like cells. SHSY5Y cells differentiated for 7 days with 10 um retinoic acid were treated with 50–400 pM of recombinant HIV-1 protein from the X4-tropic IIIB or R5-tropic BaL or SF162 strains for 48–72 h before immunofluorescent staining for β-III-tubulin. Data shown are the average of five independent assays per group. GraphPad Prism was used to determine statistical significance by one-way ANOVA and subsequent Tukey’s test compared with control as indicated. Arrows indicate shortened axons, and stars show axons with increased length. Quantification of axonal length. Values represent the mean ± SD of axonal length (μM), calculated from measurement of 20 axons per group. Tukey’s test or Dunn’s test compared with control. **a** Control. **b** IIIB Env. **c** BaL Env. **d** None–IIIB200, *p* = .0187; none–IIIB400, *p* = .0032; IIIB50–100, *p* = .029; IIIB50–200, *p* = .0014; IIIB50–400, *p* = .0002. **e** None–BaL100, *p* = .0335; none–BaL 200, *p* = .0011; none–BaL 400, *p* = .0002; BaL50–400, *p* = .0444. **f** None–SF162 200, *p* = .0023; none–SF162 400, *p* < .0001; SF162 50–400, *p* = .0011; SF162 100–400, *p* = .0083. **g** None–IIIB50, *p < .0001;* IIIB50–BaL50, *p = 0.0092;* IIIB50–SF162 50, *p* = 0.0028. **h** None–BaL100, *p* = 0.0005; None–SF162 100, *p* = 0.0497*.***i** None–BaL200, *p* < .0001; None–SF162 200, *p* = 0.0036; IIIB200–SF162 200, *p* = 0.0015. **j** None–BaL400, *p* < .0001; none–SF162 400, *p* < .0001; IIIB400–BaL 200, *p* = 0.0002; IIIB400–SF162 200, *p* = 0.0002

With the availability of suppressive antiretroviral therapy, cases of HIV-associated dementia (HAD) involving loss of neurons are far less prevalent. However, a large percentage of HIV-infected individuals continue to experience cognitive and/or neuropsychiatric comorbidities (neuroHIV) that negatively impact daily living activities (Saylor et al., 2016). Based on the findings of elevated inflammatory mediators in the plasma and cerebrospinal fluid (CSF), ongoing cellular activation is a suspected player, but the mechanisms remain to be identified (Spudich 2016). The type of neuronal injury seen now in neuroHIV more commonly involves damage to synaptic connections between neurons and alterations in dendritic growth and arborization (Ellis et al. 2007). Past work has shown that IIIB gp120 envelope (IIIB Env) protein, which enables HIV to enter cells via the CD4-CXCR4 (X4) pathway, damages synaptic connections (Kim et al., 2011). Whether elevated osteopontin (OPN) in the brain parenchyma plays a role in this process is not known. We first utilized the SHSY5Y human neuroblastoma cell line differentiated to more neuronal-like cells with retinoic acid (RA) to establish an in vitro model to investigate the impact of HIV Envs in the absence and presence of osteopontin on axonal morphology and potential mechanisms. We also included Env proteins from HIV strains (BaL and SF162), known to infect and replicate in brain astrocytes and myeloid cells through the CD4-CCR5 (R5) receptors. RA-differentiated SHSY5Y cells were treated with 50–400 pM IIIB for 48–72 h, followed by immunostaining for anti-β-III-tubulin which allowed visualization of the entire neuronal cytoskeleton. Incubation of SHSY5Y cells with 200–400 pM IIIB Env induced a significant decrease of the mean length of axons leading to the appearance of damaged and shortened axons, compared with the control (Fig. 1A, B, D). Unexpectedly, and in contrast to IIIB Env, we found that in cultures treated with 100–400 pM BaL or SF162 Env, the mean length of axons was significantly increased (Fig. 1C, Fig. 1E-1J).

**Fig. 2:**
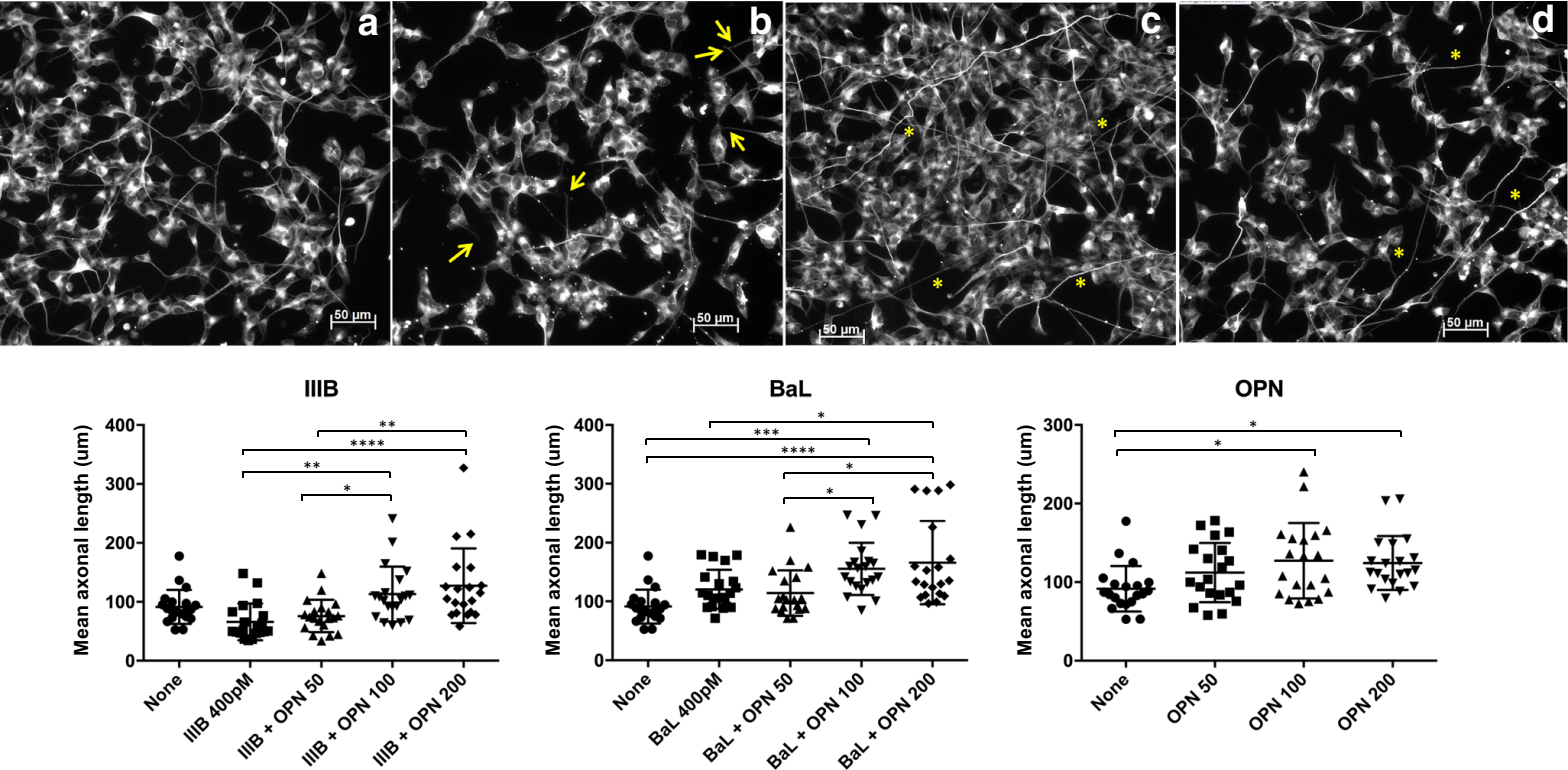
OPN reverses HIV IIIB gp120–induced decreases in neurite length and potentiates the impact of R5-tropic Env in retinoic acid–differentiated SHSY5Y neuronal-like cells. SHSY5Y cells were differentiated, treated, subjected to IF, and analyzed as described in Figure legend 1 in the presence or absence of OPN at 50–200 ng/ml and/or with HIV BaL Env or IIIB gp120 at 400 pM as indicated. Arrows indicate shortened axons, and stars show axons with increased length. GraphPad Prism was used to determine statistical significance by one-way ANOVA and subsequent Tukey’s test or Dunn’s test compared to control (parametric, OPN, Fig. 2g) or Krusalis-Wallis (non-parametric, IIIB, Fig. 2e). Representative images of treated cells stained for β-III-tubulin: **a** control, **b** IIIB only, **c** BaL and OPN 100 ng/ml, **d** OPN 100 ng/ml. **e** IIIB: IIIB–OPN100, *p* = .0049; IIIB–OPN200, *p* = .0001; IIIBOPN50–OPN100, *p* = .0452; IIIBOPN50–OPN200, *p* = .0018. **f** BaL: none–BaLOPN100, *p* = .0002; none–BaL OPN200, *p* < .0001; BaLOPN50–BaLOPN100, *p* = .0412; BaLOPN50–BaLOPN200, *p* = .0046; BaL–BaLOPN200, *p* = .0182. **g** OPN: none–OPN100, *p* = .0198; none–OPN200, *p* = .0377

OPN reverses HIV IIIB gp120-induced axonal damage in retinoic acid–differentiated SHSY5Y neuronal-like cells. We then examined the effect of OPN on neuronal morphology to determine whether it potentiated or blocked IIIB Env–induced damage. Interestingly, RA-differentiated SHSY5Y cells treated with a combination of IIIB gp120 and OPN at 100 and 200 ng/mL had significantly increased neurite growth and longer axonal lengths, in contrast to the decrease observed after exposure of SHSY5Y cells to IIIB alone (Fig. 2B, E). Exposure of RA-differentiated SHSY5Y cells to BaL Env (400 pM) and OPN (50–200 ng/mL) resulted in a dose-dependent increase of axonal length compared with the untreated and BaL only controls (Fig. 2E). Treatment with OPN alone, at 100 and 200 ng/mL, was sufficient to significantly increase axonal length, compared with the untreated control (Fig. 2C and F). These results suggest that in addition to promoting neurite growth, OPN is able to block the decrease of axonal length induced by IIIB Env.

### Osteopontin (OPN), in a dose-dependent manner, blocks HIV_IIIB_ Env inhibition of neurite growth and significantly increases the expression of dendrites in cultured primary rat cortical neurons

**Fig. 3:**
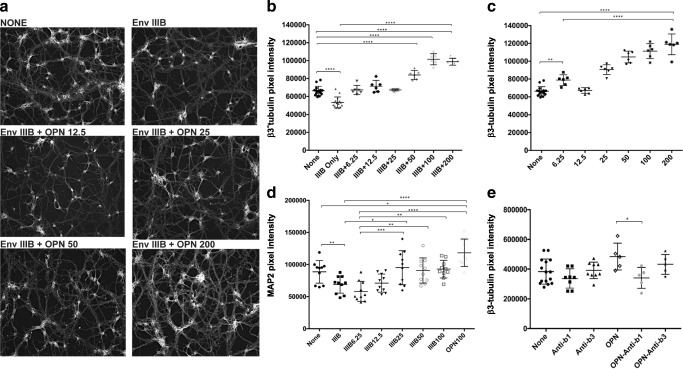
Osteopontin (OPN), in a dose-dependent manner, blocks HIV_IIIB_ Env inhibition of neurite growth and significantly increases the expression of dendrites in cultured primary rat cortical neurons. **a**, **b**, **c**, **d** E18 primary rat neurons isolated from the prefrontal cortex were differentiated for 4–5 days before treatment with increasing concentrations of OPN (6.25 pM, 12.5 pM, 25 pM, 50 pM, 100 pM, or 200 pM) in the presence or absence of 400 pM HIV IIIB envelope as indicated and stained and quantified for either β-III-tubulin or MAP2 expression. **a** Representative images of stained neurons. **b** None–IIIB, *p* < .0001; none–IIIB50, *p* < .0001; none–IIIB100, *p* < .0001; none–IIIB200, *p* < .0001; IIIB only–IIIB6.25, *p* < .0001; IIIB only–IIIB12.5, *p* < .0001; IIIB only–IIIB25, *p* < .0001; IIIB only–IIIB50, *p* < .0001; IIIB only–IIIB100, *p* < .0001; IIIB only–IIIB200, *p* < .0001; IIIB6.25–IIIB50, *p* < .0001; IIIB6.25–IIIB100, *p* < .0001; IIIB6.25–IIIB200, *p* < .0001; IIIB12.5–IIIB50, *p* = .0029; IIIB12.5–IIIB100, *p* < .0001; IIIB12.5–IIIB200, *p* < .0001; IIIB25–IIIB50, *p* < .0001; IIIB25–IIIB100, *p* < .0001; IIIB25–IIIB200, *p* < .0001; IIIB50–IIIB100, *p* < .0001; IIIB50–IIIB200, *p* = .0002. **c** None–OPN6.25, *p* = .0053; none–OPN25, *p* < .0001; none–OPN50, *p* < .0001; none–OPN100, *p* < .0001; none–OPN200, *p* < .0001; OPN6.25–OPN50, *p* < .0001; OPN6.25–OPN100, *p* < .0001; OPN6.25–OPN200, *p* < .0001; OPN12.5–OPN25, *p* < .0001; OPN12.5–OPN50, *p* < .0001; OPN12.5–OPN100, *p* < .0001; OPN12.5–OPN200, *p* < .0001; OPN25–OPN50, *p* = .0101; OPN25–OPN100, *p* < .0001; OPN25–OPN200, *p* < .0001; OPN50–OPN200, *p* = 0.106. **d** None–IIIB6.25, *p* = .0087; none–OPN100, *p* = .0119; IIIB–IIIB25, *p* = .0402; IIIB–OPN100, *p* < .0001; IIIB6.25–IIIB25, *p* = .0006; IIIB6.25–IIIB50, *p* = .0038; IIIB6.25–IIIB100, *p* = .0017; IIIB6.25–OPN100, *p* < .0001. **e** Blocking β1-integrin receptor interferes with OPN modulation of β3-tubulin levels in cultured primary rat cortical neurons. Differentiated rat cortical neurons were treated with inhibitory antibodies against β1- or β3-integrin in the absence or presence of 100 ng/ml OPN and the level of β-III-tubulin expression quantified at 48 h post-treatment. OPN-anti-β1, *p* = .0407

The cytoskeleton of primary neurons is highly differentiated with each cell containing an axon and an intricate dendritic network that is not fully represented in RA-differentiated SHSY5Y cells. To determine whether the effect of OPN was recapitulated in primary cells, E18 rat cortical neurons were cotreated with HIV Env and increasing doses of OPN (6.25–200 ng/ml) in combination or alone for 48 h followed by quantification of neurite growth by immunofluorescent staining for β3-tubulin. OPN at the lowest dose blocked HIV envelope–induced neurite loss and promoted neurite growth in a dose-dependent manner (Fig. 3A and B). Moreover, staining and quantification of dendrites using fluorescent labeling of microtubule-associated protein 2 (MAP2) revealed that OPN at higher doses significantly blocked HIV envelope decreases in MAP2 levels (Fig. 3D).

### Blocking β1-integrin receptor interferes with OPN modulation of β-III-tubulin levels in cultured primary rat cortical neurons

OPN can be secreted by cells and contains an Arg-Gly-Aspartic acid (RGD) motif near its amino-terminal end that allows it to bind and bidirectionally transduce signals through specific beta-integrin receptors including β1 and β3 (Hynes 2002). Functional antibodies with well-described inhibitory activity against these integrins (Hynes 2002, Charrier et al., 2010, Ning et al., 2013) were used to determine whether OPN engagement with these receptors was involved in its ability to alter neurite growth. Cotreatment of neuronal cultures with anti-integrin-functioning antibodies had no effect on β-III-tubulin levels in cortical cells in control untreated wells (Fig. 3E). In contrast, in OPN-treated neurons, co-incubated with an antibody against β1-integrin, β-III-tubulin levels were significantly decreased (Fig. 3E). While engagement of β3-integrin receptors could partially reverse OPN’s ability to increase β3-tubulin levels, the difference was not significant (Fig. 3E). These results suggest that OPN signaling through β1-integrin is required for stimulation and maintenance of neurite growth.

### OPN-induced increases in cortical neurite expression in the presence or absence of HIV IIIB Env are blocked by the mTOR inhibitor rapamycin

**Fig. 4:**
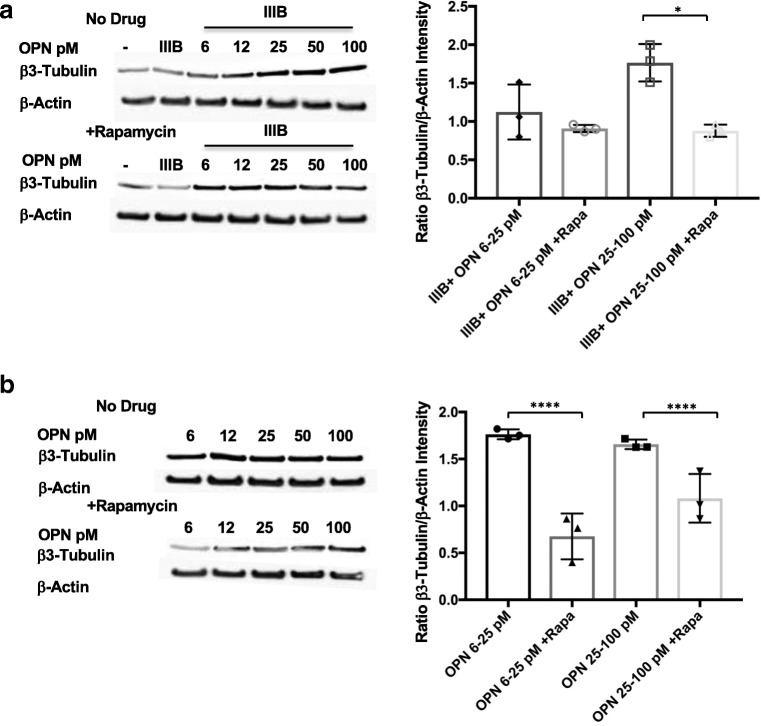
OPN-induced increases in cortical neurite expression in the presence or absence of HIV IIIB Env are blocked by the mTOR inhibitor rapamycin. Differentiated rat cortical neurons in poly-D-lysine-coated 24-well plates were treated in triplicate with increasing concentrations of OPN (6.25 pM, 12.5 pM, 25 pM, 50 pM, 100 pM, or 200 pM) in the presence or absence of 400 pM HIV IIIB envelope and rapamycin (20 nM) as indicated. To obtain sufficient protein for western analyses, the triplicate wells were pooled and 1–2 μg of protein was loaded onto 4–12% Bis-Tris gradient gels for SDS-PAGE and western blot analyses for quantification of β-III-tubulin and β-actin expression. **a** IIIB25–100, *n* = 3, *p* = .0193; **b** OPN6–25 w/wo rapamycin, *n* = 3, *p* = .0004. OPN25–100 w/wo rapamycin, *p* = .0199

The mammalian target of rapamycin (mTOR) pathway has been identified as a pathway involved in the dynamic regulation of neuronal cytoskeleton morphogenesis (Switon et al., 2017). To determine whether mTOR signaling was involved in OPN’s effects on cortical neurite expression, cells were treated in triplicate wells in a 24-well plate with HIV IIIB Env and increasing doses of OPN in the presence or absence of 20 mM rapamycin, a well-validated inhibitor of mTOR signaling. In order to obtain sufficient protein for analyses, triplicate wells were pooled and subjected to western blot analyses and quantified for β3-tubulin expression. At higher concentrations of OPN (25–100 ng/ml), rapamycin treatment significantly blocked OPN-induced dose-dependent increases in β-III-tubulin expression (Fig. 4A). Maintenance of β-III-tubulin levels in cortical neurons treated only with OPN was significantly inhibited by rapamycin at both lower and higher concentrations of OPN (Fig. 4B). These results suggest that mTOR signaling is required for OPN-induced increases in β-III-tubulin expression.

### In HIV envelope–treated rat cortical neurons, low, but not higher, levels of OPN activate mTORC1

**Fig. 5:**
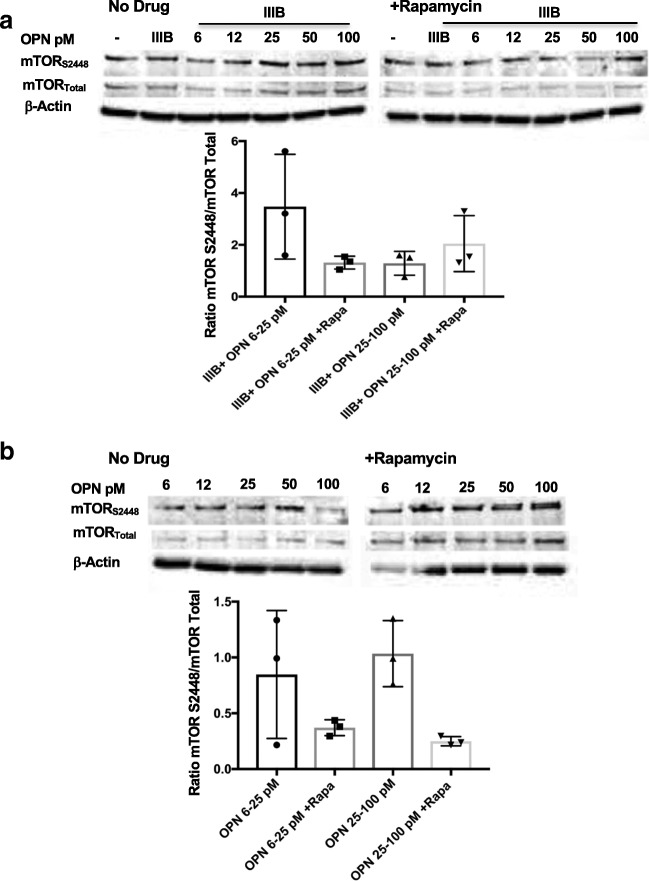
In HIV envelope–treated rat cortical neurons, low, but not higher, levels of OPN activate mTORC1**.** Differentiated rat cortical neurons were prepared and treated as described in figure legend 4 and subjected to western analyses for mTOR S2448 and mTOR total protein expression. The differences were not significant

**Fig. 6:**
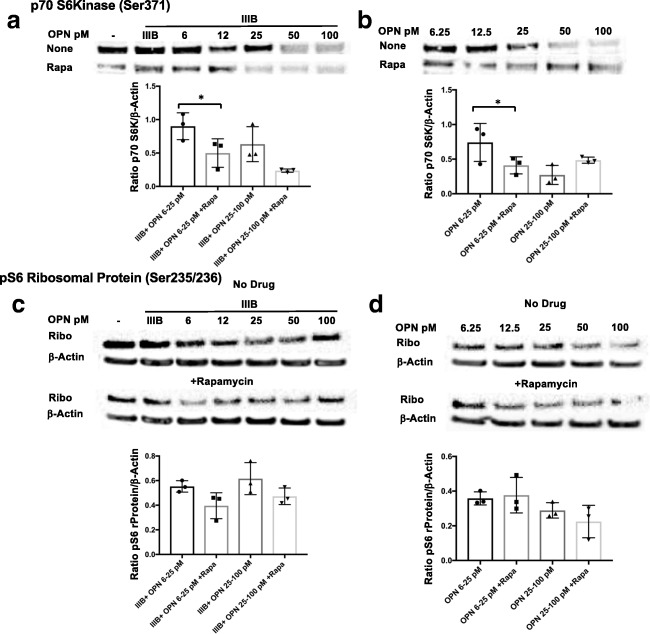
Activation of p70 S6 kinase, a downstream target of mTORC1 signaling by low doses of OPN in the presence or absence of HIV Env in cotreated cortical neurons. Differentiated rat cortical neurons were prepared and treated as described in figure legend 4 and subjected to western analyses for p70 S6 kinase (Ser371), pS6 ribosomal protein (Ser235/236), and β-actin expression. **a** IIIB6.25–25, with/without rapamycin *n* = 3, *p* = .0138; **b** OPN6–25 w/wo rapamycin, *n* = 3, *p* = .0355

Activation of the mTOR pathway results in the phosphorylation of mTORC1 or mTORC2 and specific downstream target proteins. Western blot analyses as delineated above were performed for mTOR and two downstream targets of the mTORC1 complex. In HIV IIIB Env and OPN cotreated cortical neurons, there was an increased mTOR phosphorylation at Ser2448, with exposure to low levels (6.5–12.5 ng/ml) of OPN that was inhibited by rapamycin, and no effect at higher concentrations of OPN (25–100 ng/ml) (Fig. 5A). In contrast, the level of mTOR Ser2448 phosphorylation was not modulated by sole treatment with OPN (Fig. 5B). While rapamycin could partially inhibit this, the differences were not significant (Fig. 5B). Analyses of the activation of mTOR downstream target p70S6 kinase on Ser371 showed a trend that in HIV IIIB Env–treated cortical neurons, p70S6K was inhibited with increasing concentrations of OPN (Fig. 6A). Interestingly, a similar pattern was observed in cortical neurons treated with increasing does of OPN (Fig. 6B). We next probed for the downstream target of p70S6 kinase and pS6 ribosomal protein, the activation of which can be detected by phosphorylation at S235/236. In HIV IIIB Env–treated cortical cultures exposed to increasing doses of OPN, compared with untreated cells, the phosphorylation of this protein was not appreciably modulated by OPN and was decreased by 50% by rapamycin (Fig. 6C). A similar pattern of regulation in the absence or presence of rapamycin was seen in cortical neurons treated solely with increasing doses of OPN (Fig. 6D), suggesting that modulation of pS6 ribosomal protein was not involved in OPN-mediated neurite growth enhancement. Taken together, these results suggested the need for additional probing to identify the rapamycin-sensitive pathway involved.

### The mTORC2 complex is activated in HIV Env-OPN-cotreated cortical neurons

**Fig. 7:**
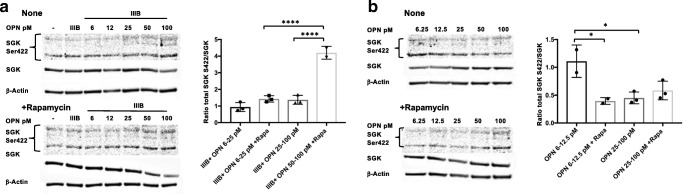
Activation of the downstream mTORC2 substrate, stress-glucocorticoid kinase 1 (SGK1) in HIV Env-OPN-cotreated cortical neurons. Differentiated rat cortical neurons were prepared and treated as described in figure legend 4 and subjected to western analyses for stress- and glucocorticoid-regulated kinase-1 (SGK1) Ser422, SGK1 total protein, and β-actin expression. **a** IIIB25–100, with/without rapamycin *n* = 3, *p* < .0001; and rapamycin-IIIB6.25–25 and rapamycin-IIIB50–100, *n* = 3, *p* < .0001. **b** OPN6–25 w/wo rapamycin, *n* = 3, *p* = .0202; OPN6–25 and OPN25–100, *p* = .0192

**Fig. 8:**
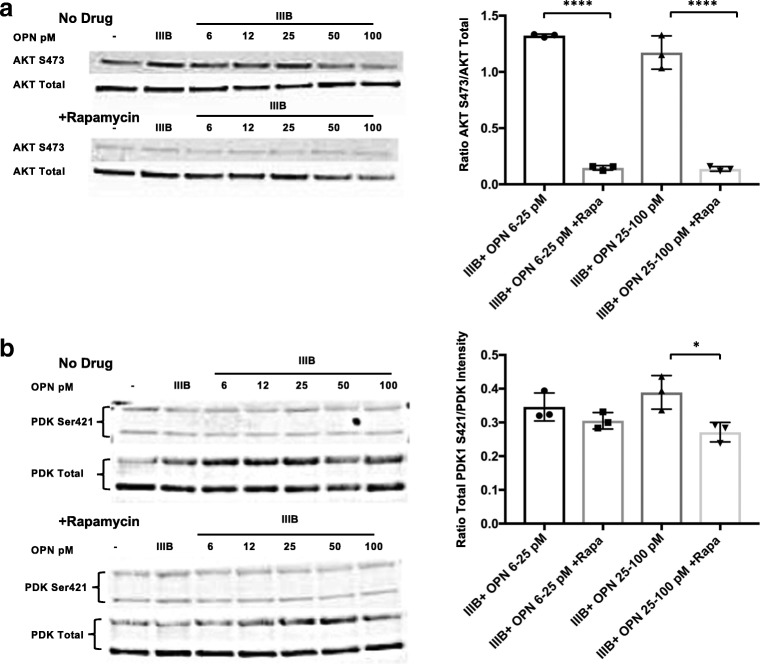
AKT phosphorylation is activated via an mTORC2-mTORC1 pathway interaction, in a PDK-independent manner. Differentiated rat cortical neurons were prepared and treated as described in figure legend 4 and subjected to western analyses for the expression of protein kinase B (Akt Ser473), total Akt protein, phosphoinositide-dependent kinase-1 (PDK1 Ser421) and total PDK protein levels. **a** IIIB6.25–25, with/without rapamycin *n* = 3, *p* < .0001; IIIB25–100, with/without rapamycin *n* = 3, *p* < .0001. **b** IIIB25–100, with/without rapamycin *n* = 3, *p* = .0207

**Fig. 9:**
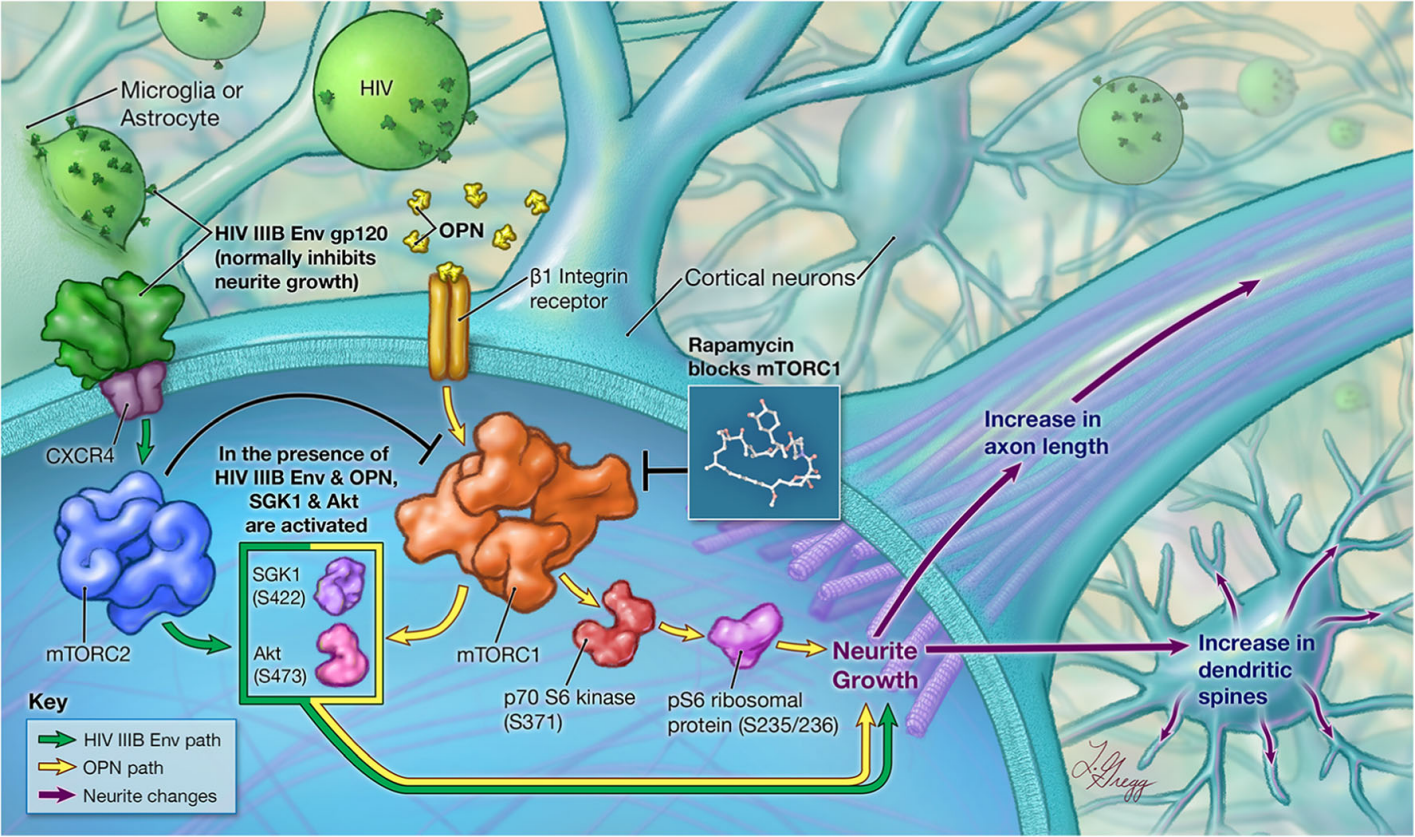
Osteopontin (OPN)-induced β1-integrin activation and HIV-1 envelope signaling converge on mTORC1/2 pathways in a mechanism that promotes neurite outgrowth. HIV X4-tropic envelope protein is a well-known neurotoxin. A novel phenotype of increased neurite growth was observed in primary rat cortical neurons exposed to increasing doses of osteopontin in the presence or absence of HIV X4-tropic envelope. Osteopontin promoted neurite growth as assessed by increases in b3-tubulin and MAP2 staining. Increases in osteopontin-mediated b3-tubulin expression were inhibited by rapamycin, a well-known inhibitor of mTORC1, thus implicating this pathway in the mechanism of action. The signaling cascade can be initiated through β1-integrin receptors, as functional antibody blocking studies abrogate the effect of osteopontin. A modest level of activation of downstream mTORC1 substrate p70 S6 kinase was found suggesting activation of mTORC1. However, in the presence of HIV IIIB envelope and osteopontin, SGK1 and Akt, both established targets of mTORC2 signaling, are activated. Interestingly, the activation of SGK1 in neurons cotreated with HIV IIIB envelope and higher levels (50–100 pM) of osteopontin are insensitive to rapamycin inhibition confirming the activation of mTORC2. In contrast, low levels of osteopontin (6–12.5 pM) induce SGK activation in a manner that depends on mTORC1 signaling. Collectively, our data suggest that there is a feedback loop operating between mTORC1 and mTORC2, the outcome of which is increases in neurite growth. The mTORC1 and mTORC2 pathways as well as signaling via β1-integrin receptors have been implicated in the regulation of actin cytoskeletal dynamics, learning and memory, axon guidance, and synaptic plasticity. Therapeutic strategies which can modulate osteopontin expression in the central nervous system in the context of neuronal dysfunction may be advantageous in addressing deficits accompanying neurodegenerative processes. Image rights: © 2018 Johns Hopkins University, by Lydia Gregg

It is known that mTORC2 can inhibit mTORC1 activation using specific feedback mechanisms. To test whether mTORC2 might be activated in this experimental model, we performed western blot analyses for its downstream target, stress-glucocorticoid kinase 1 (SGK1). In the presence of HIV Env, the phosphorylation of SGK1 on Ser422 was increased with higher levels of OPN (50–100 ng/ml) and, interestingly, was potentiated by rapamycin treatment (Fig. 7A). In the absence of HIV Env, treatment of rat cortical neurons with increasing doses of OPN led to a significant reduction of SGK phosphorylation that at the highest doses was insensitive to rapamycin (Fig. 7B). Collectively, these results suggested the possibility that HIV Env signals through mTORC2, while low OPN acts via an mTORC1 pathway. We tested this idea by probing for the phosphorylation of AKT at Ser473, a well-known indicator of mTORC2 activation. Treatment of rat cortical neurons with HIV Env in the presence of OPN revealed that phosphorylated AKT Ser473 was constitutively expressed in a manner that was sensitive at each dose to strong inhibition by rapamycin (Fig. 8A). Alternatively, AKT can also be phosphorylated by phosphoinositide-dependent kinase 1 (PDK1) and blocked signaling to mTORC1 via pathways involving PRAS40 or Rheb. In western analyses of HIV IIIB Env and OPN cotreated rat cortical neurons, no dose-dependent modulation of PDK1 phosphorylation at S421 was detected (Fig. 8B). These results suggest that in HIV envelope-OPN-exposed neurons, mTORC2 activation triggers constitutive phosphorylation of AKT in a signaling cascade that drives increased neurite growth (Fig. 9).

## Discussion

In molecular screens, to identify genes that are upregulated in neurodegenerative disorders, osteopontin (OPN), and in different experimental paradigms of neuronal injury in many cases, has been identified (Brown 2012). However, the function of OPN in the central nervous system (CNS) in many of these disorders remains largely unknown. In this regard, in a model of axotomy, OPN in combination with insulin growth factor 1 facilitated axon regeneration in the retinal ganglion (Duan et al., 2015). Additionally, in models of stroke and brain injury, the induction of OPN expression served a pro-survival function (van Velthoven et al., 2011). Although our initial observation of enhanced neurite growth was made using a neuronal-like cell line treated with recombinant human OPN, the phenotype was recapitulated in primary rat cortical neurons treated with recombinant rat OPN. Rat neurons have been shown to possess functional characteristics that are very similar to human neurons, and therefore, primary cultures of these cells have served as useful experimental models for mechanistic studies (Semple et al., 2013). Indeed, we found that the use of species-matched recombinant OPN was required, indicating that OPN must interact with and signal through its cognate receptors on differentiated human SHSY5Ys and rat cortical neurons, respectively. Using an in vitro primary rat cortical neuron model in which we could expose cells to HIV IIIB Env and increasing levels of OPN, we found that rapamycin, a well-known inhibitor of mTORC1 signaling, blocked increases in neurite growth (see Model in Fig. 9). Further analyses of downstream targets of mTORC1 showed that low levels of OPN activated this pathway, but it was not sustained at higher doses in the presence of HIV IIIB Env (Figs. 5, 6). Rather, in the presence of HIV IIIB Env, at higher doses of OPN, SGK1, a downstream target of mTORC2, became activated in a rapamycin-insensitive manner (Fig. 7). SGK1 has been previously implicated as a key regulator of several important neuroprotective pathways, learning, and memory, and as an effector in the modulation of neuronal cytoskeletal dynamics (Lang et al., 2010). The mTOR pathways are required for regulating critical cellular growth pathways including autophagy, proliferation, survival, and lipid synthesis (Hong and Inoki 2017). Monitoring of these different cellular states involves feedback promotion and inhibition between mTORC1 and mTORC2, and Akt is a central player in this loop. It is currently accepted that activation of Akt (Ser473) can occur through a PI3K-dependent pathway involving PDK1 or via the mTORC2 complex (Saxton and Sabatini 2017). Our results show that Akt is activated by mTORC2, but not through PDK1 (Fig. 8). Interestingly, the activation of Akt is strongly inhibited by rapamycin. Through its association with the FKBP12 protein, rapamycin is a specific inhibitor of mTORC1, but with high levels of the drug (200 nM-1 uM) (Toschi et al., 2009) and prolonged exposure, mTORC2 can also be inhibited (Jaworski et al., 2005). The lack of inhibition of SGK1 by rapamycin in our model suggests that the mTORC2 pathway was active, and rather that an intricate feedback loop between mTORC1 and mTORC2 is operant, resulting in an outcome that counteracts the injurious impact of HIV IIIB Env and promotes neurite growth (Fig. 9). In this regard, the participation of both mTORC1 and mTORC2 in the modulation of actin cytoskeletal dynamics in neurons has been implicated in the control of dendritic arborization in hippocampal neuronal cultures (Urbanska et al., 2012). Collectively, our results support a model in which HIV IIIB Env engagement of its receptor CXCR4 and OPN signaling through β1-integrins synergize to activate an mTORC2-dependent pathway involving SGK1 and Akt that promotes neurite growth (model, Fig. 9). Interestingly, a recent report examining a model for neural stem cells survival suggests the possibility that OPN and CXCR4 signaling pathways intersect (Rabenstein et al., 2015). Additionally, the role of integrins as facilitators of inside-out signaling from the extracellular matrix to the nucleus has long been known. In the CNS, at least ten different integrin heterodimers are expressed where they play key roles in neuron and neuron-glia synapse morphogenesis and maturation (Pinkstaff et al., 1999, Chavis and Westbrook 2001, Shi and Ethell 2006), long-term potentiation (LTP), spatial memory, and the regulation of excitatory synaptic strength (Staubli et al., 1990, Chan et al., 2006, Huang et al., 2006, Kramar et al., 2006).

Axonal injury has been shown to be the first step in HIV-mediated neuronal degeneration, which then spreads to the cell body followed by neuronal apoptosis (Ellis et al. 2007; Mocchetti et al. 2012). Despite the predominance of macrophage-tropic, R5 strains of HIV-1 in the brain of infected individuals (Gorry et al., 2001, Peters et al., 2004, Gonzalez-Perez et al., 2012), most studies have used the X4-tropic Env of HIV IIIB to determine the role of this protein in inducing neuronal damage. In those studies, examining cell death mediated by X4 IIIB and R5 SF162 gp120, ERK and p38 MAPK signaling pathways were activated (Kaul et al., 2007, Medders et al., 2010). A previous study from Bachis showed that R5-utilizing HIV BaL gp120 microinjected into the rat striatum was less cytotoxic than X4 Env (Bachis et al., 2010). While we observed that OPN was also able to protect neurons from excitotoxicity (data not shown), an unexpected and additional novel finding in our study is the ability of R5 clade B Envs to promote neurite growth. OPN was able to reverse the inhibition of X4 IIIB Env on axonal length suggesting the possibility that it may activate a downstream factor that lies in the same pathway triggered by R5 Env. In this regard, a recent finding showed that OPN applied on the growth surface as a substrate could stimulate neurite growth through β1-integrin and CD44 receptors (Plantman 2012). Moreover, OPN, by binding to integrins, can activate Rac-1 GTPase signaling cascades leading to actin remodeling and cytoskeletal rearrangements (Kang et al., 2008). Indeed, integrin signaling (Ivins et al., 2000) and Rac GTPases in the control of actin cytoskeletal dynamics are also known to play a crucial role in neurite growth (Luo 2002, Ng et al., 2002). To stimulate migration of trophectoderm cells, OPN was shown to engage the mTOR pathway via signaling using β1- and β3-integrin receptors leading to the induction of focal adhesion assembly (Kim et al., 2010). Interestingly, CCR5 ligands MIP-1α and RANTES have also been shown to stimulate neuroprotective pathways (Meucci et al., 1998, Kaul et al., 2007, Bachis et al., 2010). Collectively, these findings suggest that in an attempt to counteract HIV-mediated neuronal injury, multiple cellular signaling pathways are stimulated in neurons and, based on our prior work, in glia cells as well (Brown et al., 2011, Silva et al., 2015).

An optimal level of OPN released by the host is likely beneficial in reducing neuronal injury induced by HIV in the CNS. Our finding that OPN activates mTOR function is important because this pathway in the brain is intimately involved in controlling the mechanisms of learning and memory (Jaworski et al., 2005). The function of mTORC1 and/or mTORC2 in long-term potentiation (LTP), long-term depression (LTD) (Zhu et al., 2018), learning and memory, neuronal survival, differentiation, and morphogenesis has been reported (Switon et al., 2017). Moreover, specific extracellular factors can activate the mTOR pathway including growth factors, hormones, brain-derived nerve growth factor, neurotransmitters, GABA, monoamines, acetylCoA, and neuropeptides (Hong and Inoki 2017). Our findings that extracellular OPN blocks the negative effects of HIV Env on neurite growth via an integrin-mTORC1/2 signaling pathway has implications for our understanding of ongoing neuronal injury and cognitive dysfunction in the CNS of individuals on suppressive antiviral therapy. In this regard, therapeutic strategies, which can modulate OPN expression in the CNS, may be advantageous in counteracting the ongoing injury and memory impairment related to HIV-1 reservoirs in the brain and spinal cord.
